# Overexpression of RANKL by invariant NKT cells enriched in the bone marrow of patients with multiple myeloma

**DOI:** 10.1038/bcj.2016.108

**Published:** 2016-11-11

**Authors:** E Spanoudakis, M Papoutselis, E Terpos, M A Dimopoulos, C Tsatalas, D Margaritis, A Rahemtulla, I Kotsianidis, A Karadimitris

**Affiliations:** 1 Department of Haematology, Democritus University of Thrace, Alexandroupolis, Greece; 2 Department of Clinical Therapeutics, University of Athens School of Medicine, Athens, Greece; 3 Centre for Haematology, Department of Medicine, Hammersmith Hospital, Imperial College London, London, UK

Upregulation of soluble or membrane bound receptor activator of nuclear factor kappa B ligand (RANKL) in the multiple myeloma (MM) microenvironment is pivotal for the pathological activation of osteoclasts and development of myeloma bone disease.^1^ This process largely depends on the ability of malignant plasma cells to provoke apoptosis of osteocytes.^2^ Highlighting the importance of RANKL in the biology of MM, it was previously shown that the RANKL to osteoprotegerin (a RANKL decoy receptor) ratio predicts survival.^3^ RANKL is also induced on the surface of CD4^+^ T cells by IL-7 (ref. 4) and CD4^+^ Th17 T cells with pro-osteoclastogenic activity are increased in the peripheral blood (PB) and bone marrow (BM) of patients with myeloma.^5, 6^

Invariant NKT (iNKT) TCRVα24Jα18/Vβ11 cells are a small but powerful subset of CD1d-restricted, glycosphingolipid (GSL)-specific immunoregulatory T cells that can regulate a variety of immune responses,^7^ including anti-tumour immunity, hematopoiesis and osteoclast differentiation and activation.^8^ iNKT cells are dysfunctional in MM in that they display reduced ability to secrete interferon-gamma thus contributing to myeloma immune evasion.^9^ CD1d downregulation in myeloma plasma cells in advanced disease^10^ represents another aspect of subversion of the CD1d-iNKT cell axis of tumour immune surveillance in MM.

We showed previously that upon glycolipid activation, iNKT cells enhance maturation and activation of osteoclasts *in vivo* and that the iNKT cell-deficient CD1d−/− mice display a mild osteopetrotic phenotype.^11^ Further, myeloma cell-derived GSL can directly activate osteoclasts and thus in synergy with RANKL contributes to myeloma bone disease.^12^

Whether iNKT cells also contribute to the enhanced RANKL production in the MM microenvironment is not known. To address this, we studied the dynamics of RANKL expression in iNKT cells from normal individuals and from patients with MM.

To better understand the possible contribution of iNKT cells to the increase in RANKL in MM, we first established the pattern of RANKL expression in PB conventional T and iNKT cells in normal individuals (*n*=40, median age 66, range: 43–89 years; Supplementary Table 1). *Ex vivo* paired sample assessment by flow cytometry showed that expression of surface RANKL was modestly but significantly higher in normal donor iNKT cells compared with conventional T cells: mean fluorescence intensity (MFI) RANKL/isotype Ab (RANKL MFI henceforth): median 1.12 vs 1.02, respectively, (*P*=0.001, Figure 1a; see materials and methods in Supplementary File). In line with surface expression, we confirmed that purified iNKT cells (*n*=7 donors) expressed 1.66-fold more RANKL mRNA (soluble and surface) than iNKT cell-depleted conventional T cells from the same donor (*P*=0.043; Figure 1b).

To investigate whether RANKL expression in iNKT cells is also dynamically regulated in response to T cell Receptor (TCR) stimulation, negatively selected total T cells from normal donors (*n*=11) were activated with anti-CD3/CD28-covered beads followed by flow cytometric analysis at different time points. We found that compared with baseline, surface RANKL MFI on iNKT cells increased by twofold at 12 h (*P*=0.01) and 3.1-fold by 36 h (*P*<0.001; Figure 1c). RANKL expression also increased but to a lesser extent on conventional T cells by 1.2- and 1.3-fold at 12 and 36 h, respectively, (*P*=0.016 and *P*=0.013, respectively; Figure 1c).

In line with the higher expression of RANKL in iNKT cells, supernatants from CD3/CD28 bead-activated iNKT cells and in the presence of a limiting concentration of RANKL supported development of 1.5-fold more autologous monocyte-derived osteoclasts as supernatants from similarly activated, iNKT cell-depleted T cells showing that activated iNKT cells are more efficient in inducing osteoclast growth (*P*=0.04; Figure 1d).

Therefore, we establish for the first time the dynamics of RANKL expression in iNKT cells in normal individuals and we show that on per cell basis they are a richer source of surface and likely soluble RANKL than conventional T cells *ex vivo* and *in vitro*.

We next investigated RANKL expression in PB iNKT and conventional T cells of newly diagnosed, untreated MM patients (*n*=37) and patients with monoclonal gammopathy of uncertain significance (MGUS, *n*=10) or asymptomatic MM (ASM, *n*=7). Consistent with previous reports,^9, 13^ we found that the frequency of PB iNKT cells in MM patients and in MGUS/ASM patients was significantly lower than in age-matched normal controls (*n*=40): 0,06%/0,05% vs 0,17% of CD3+ cells (*P*≤0,001; Supplementary Figure 1).

However, as in normal controls, in patients with MM, we observed a small but significantly higher level of surface RANKL expression in PB iNKT than conventional T cells: RANKL MFI 1.21 vs 1.00, respectively, (*P*<0.001; Figure 2a). In MGUS/ASM patients, RANKL expression on iNKT cells in the PB was also higher compared with autologous T cells (*P*=0.02; Figure 2a). Furthermore, although surface RANKL expression on PB T cells was similar in MM patients and age-matched normal donors, it was significantly higher in iNKT cells of myeloma patients than of normal donors (*P*=0.027; Figure 2a). There was no difference on iNKT cell RANKL expression between MGUS/MM and MM patients (*P*=0.95; Figure 2a). In line with these results, purified PB iNKT cells expressed 3.52-fold more RANKL mRNA than T cells from the same patients when assessed by RQ-PCR (*P*=0.018; Figure 2b).

Finally, analysis of surface RANKL showed that MM patient iNKT cells upregulated RANKL expression upon TCR stimulation by 1.51-fold at 36 h (*P*=0.015, *n*=8; Figure 2c), whereas in T cells, RANKL levels remained unchanged (*P*=0.51; Figure 2c).

These data highlight the role of iNKT cells as a potentially important cellular source of RANKL in the MM microenvironment.

To assess RANKL expression in iNKT cells in the MM microenvironment directly, we studied iNKT cells in paired BM and PB samples (*n*=20) in untreated, newly diagnosed myeloma patients. We found that iNKT cell frequency is >10-fold higher in BM compared with PB: median 0.49% vs 0.034% (*P*<0,001; Supplementary Fig 3a and Figure 2d).

Importantly, BM iNKT cells from MM patients displayed significantly higher expression of surface RANKL compared with their PB counterparts: RANKL MFI in BM vs PB iNKT cells: median 2.57 vs 1.34 (*P*=0.031, *n*=20 paired samples; Figure 2d) suggesting that increased RANKL expression on BM iNKT cells is a tissue-specific effect. RANKL expression was also marginally higher on patient BM compared with PB-derived T cells; RANKL T cell MFI: 1.0 vs 0.99 (*P*=0.003; Supplementary Fig 3b).

We next investigated the relation between RANKL expression on PB iNKT cells and osteoclast activity as reflected by serum β-C-terminal telopeptide a sensitive and specific marker of osteoclast activity and bone resorption in patients with MM.^14^ We found that in MM patients (*n*=20), RANKL expression on iNKT cells powerfully correlated with serum β-C-terminal telopeptide levels (Pearson correlation=0.56, *P*=0.01; Supplementary Figure 2). By contrast, there was no such correlation in age-matched normal donors without evidence of osteoporosis (Pearson correlation=0.27, *n*=43, *P*=0.08; Supplementary Table 2).

Overall, both PB and BM iNKT cells from MM patients express higher levels of RANKL than PB iNKT cells from normal donors, whereas in myeloma patient, BM iNKT cells express higher levels of RANKL than BM T cells or autologous PB iNKT and T cells. Importantly, RANKL overexpression on iNKT cells of myeloma patients is strongly and dynamically related with the activity of bone resorption as assessed by β-C-terminal telopeptide levels. Patient BM is also highly enriched (>10-fold) in iNKT cells in comparison to PB, a phenomenon that is likely to be specific to myeloma since it was previously reported that in normal human BM the relative frequency of iNKT cells is similar to that of PB.^15^ This novel observation is in line with enrichment of iNKT cells in the BM of a murine model of progressive myeloma^13^ and suggests either a selective migration of iNKT cells from PB to BM or local expansion possibly in response to tumour-associated GSL presented in the context of CD1d expressed on myeloma plasma cells. Although this process might initially be important for myeloma immunosurveillance, conversion of iNKT cells phenotype into one associated with immune evasion, for example, loss of interferon-gamma secretion, it also encompasses features that are directly linked to development of myeloma bone disease. Such features include enhanced expression of RANKL in PB and especially in BM iNKT cells in patients with MM as part of a myeloma-specific dysfunctional iNKT cell phenotype that could contribute to osteoclast activation and bone destruction as well as tumour immune evasion.

The data presented here emphasize the potential role of iNKT cells in the development of myeloma bone disease and in conjunction with previous reports suggest that targeting of the CD1d-iNKT cell immune axis in MM could be a promising therapeutic strategy.^15^

## Figures and Tables

**Figure 1:**
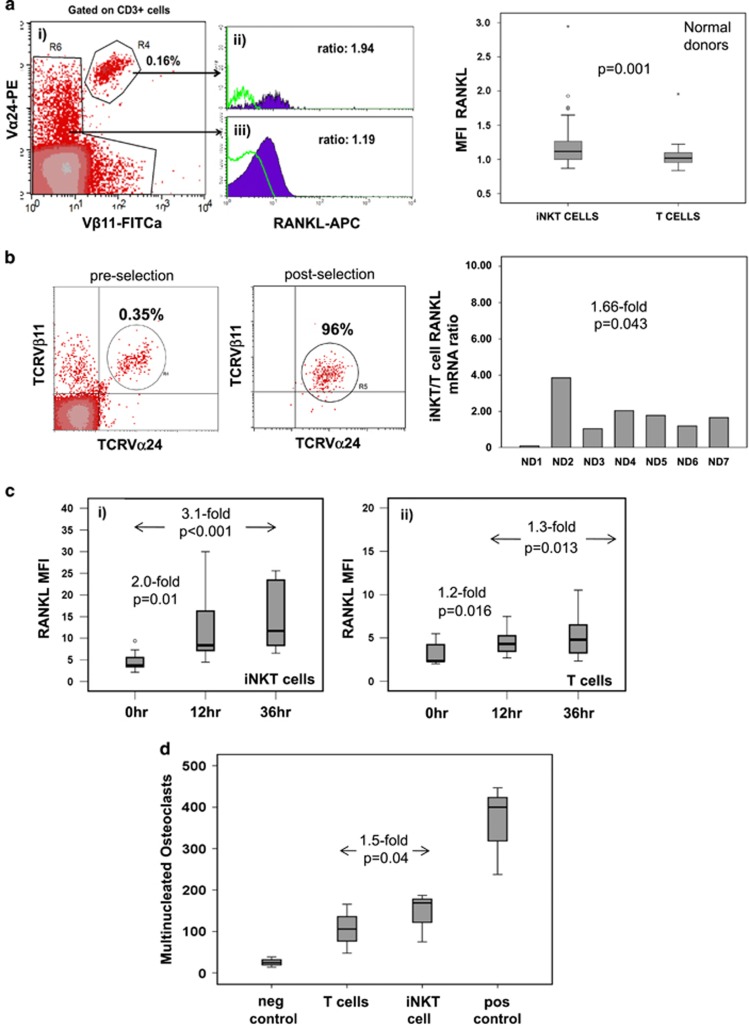
RANKL expression in PB iNKT and T cells in normal donors. (**a**) Left: surface RANKL expression in PB T and iNKT cells from normal donors as assessed by flow cytometry. (i) Identification of iNKT cells as CD3+TCRVα24+Vβ11+ cells, (ii) example of RANKL expression on iNKT cells and (iii) on non-iNKT T cells as compared with isotype control (green line) on same cell subsets. Right: cumulative data of RANKL expression on T and iNKT cells shown as MFI expression level in relation to their respective isotype control from 40 normal donors. (**b**) Left: example of iNKT cell frequency pre- and post-immunomagnetic bead selection performed with an anti-TCRVα24Jα18-specific mAb as described in materials and methods. Right: iNKT/T cell RANKL mRNA level ratio from seven normal donors. Overall, iNKT cells expressed 1.66-fold higher levels of RANKL mRNA than T cells (*P*=0.043). (**c**) RANKL expression on normal donor iNKT (left) and T (right) cells after stimulation of total T cells, including iNKT cells with anti-CD3/CD28 beads at different time points (12 and 36 h; *n*=11). Data shown are MFI. (**d**) Osteoclast formation in the presence of T and iNKT cell-derived supernatants. Positive control represents monocyte-derived osteoclasts in control cultures with M-CSF (50 ng/ml)+RANKL (25 ng/ml), negative control with M-CSF only and the other two conditions are M-CSF+RANKL (at the permissive dose of 2.5 ng/ml each) plus supernatant from activated iNKT or T cells. Osteoclasts were scored as TRAP+ cells with >3 nuclei on day 14 of the culture. Cumulative data shown as median (range) (*n*=4 normal donors, *P*=0.04 by Wilcoxon paired test).

**Figure 2:**
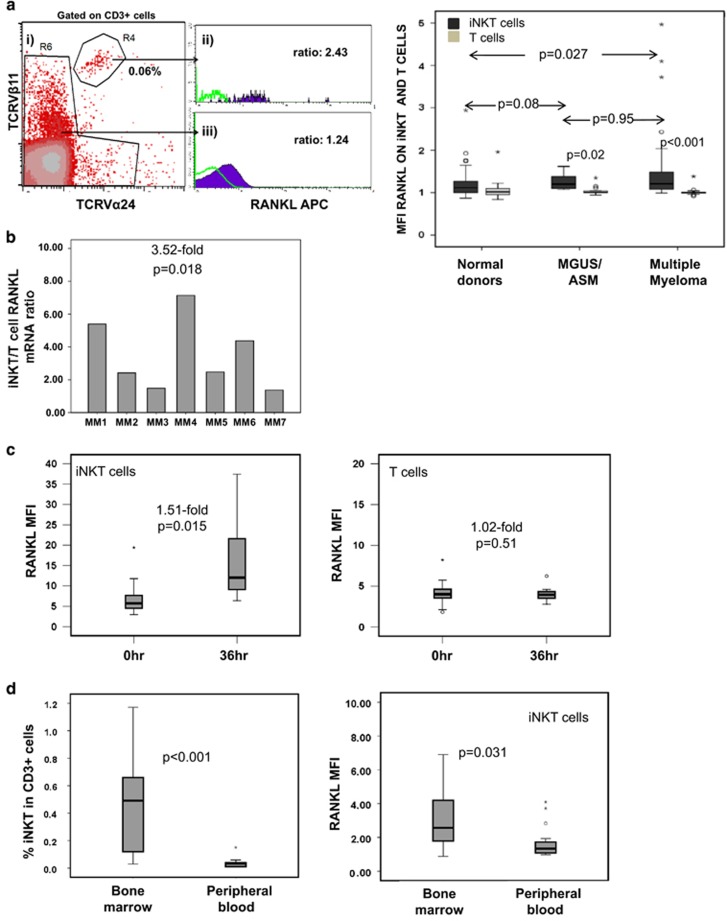
RANKL expression in iNKT and T cells in patients with MM. (**a**) Left: surface RANKL expression on PB T and iNKT cells from patients with active MM. (i) Identification of iNKT cells as CD3+TCRVα24+Vβ11+ cells, (ii) example of RANKL expression on iNKT cells and (iii) on non-iNKT T cells as compared with isotype control (green line) on same cell subsets. Right: cell surface RANKL expression on iNKT cells (dark) vs T cells (grey) in PB from patients with active MM, MGUS/ASM patients and normal donors. Higher surface RANKL expression in MM than normal donor iNKT cells, *P*=0.027, Mann-Whitney test. (**b**) RANKL mRNA levels in purified iNKT expressed in relation to paired T cell RANKL mRNA level (*n*=7 patients with active MM). Overall, iNKT cells expressed 3.52-fold higher levels of RANKL mRNA than T cells (*P*=0.018). (**c**) Cell surface RANKL expression (shown as MFI in relation to isotype control) in iNKT and T cells, respectively, from eight patients with active MM upon stimulation with anti-CD3/CD28 beads at 0 and 36 h. (**d**) Left: median frequency of iNKT cells in CD3+ cells in the BM and PB in MM patients (cumulative data; *n*=20). Right: cell surface RANKL expression in PB- and BM-derived iNKT cells from patients with active MM (*n*=20).
