# Supplementary material for: Overexpression of RANKL by invariant NKT cells enriched in the bone marrow of patients with multiple myeloma
Source: Blood Cancer J. 2016 Nov 11;6(11):e500–. doi: 10.1038/bcj.2016.108 (PMC5148055; doi:10.1038/bcj.2016.108)
Supplement: Supplementary Information [file bcj2016108x1.ppt]

## Slide 1
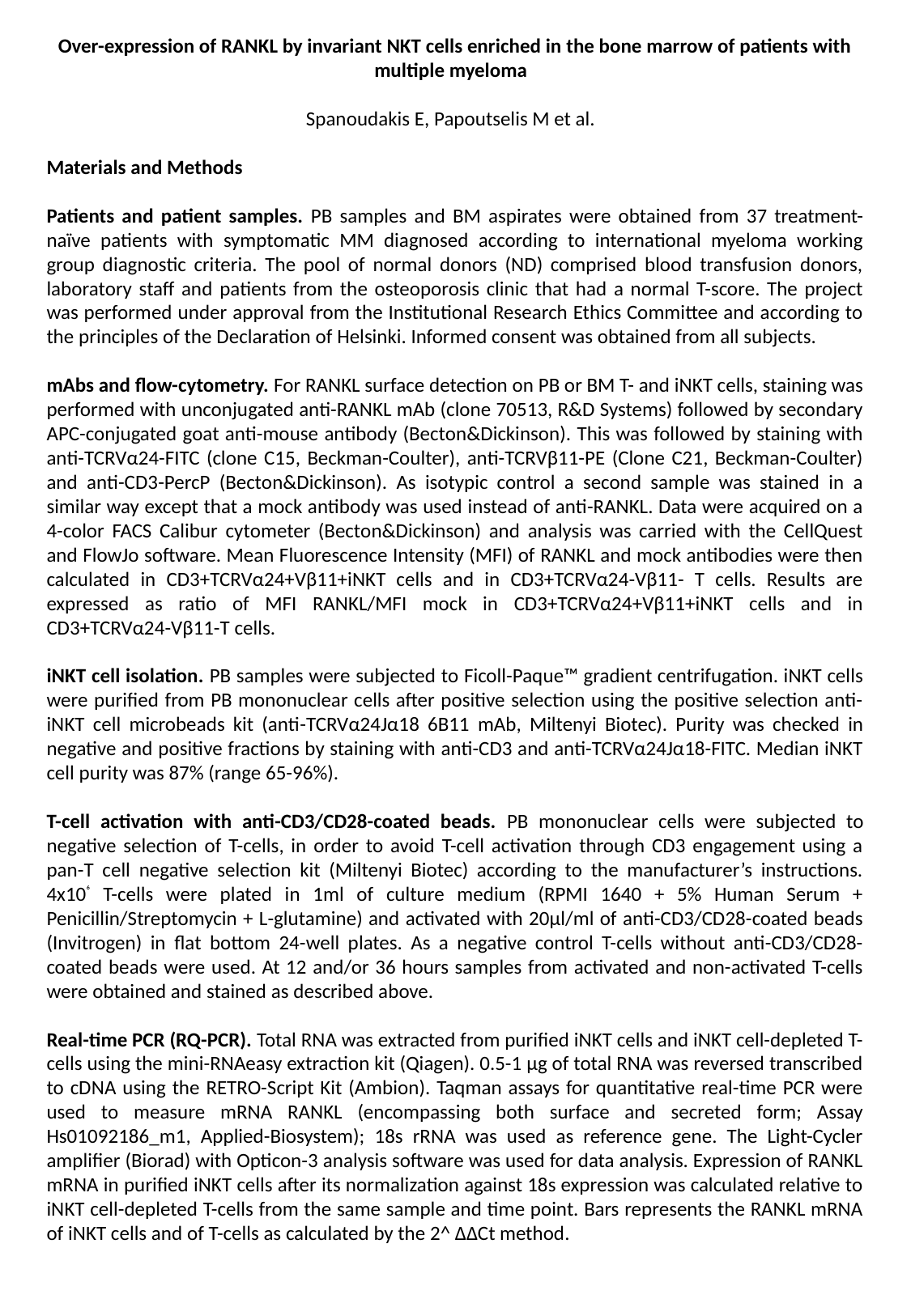

Over-expression of RANKL by invariant NKT cells enriched in the bone marrow of patients with multiple myeloma
Spanoudakis E, Papoutselis M et al.
Materials and Methods
Patients and patient samples. PB samples and BM aspirates were obtained from 37 treatment-naïve patients with symptomatic MM diagnosed according to international myeloma working group diagnostic criteria. The pool of normal donors (ND) comprised blood transfusion donors, laboratory staff and patients from the osteoporosis clinic that had a normal T-score. The project was performed under approval from the Institutional Research Ethics Committee and according to the principles of the Declaration of Helsinki. Informed consent was obtained from all subjects.
mAbs and flow-cytometry. For RANKL surface detection on PB or BM T- and iNKT cells, staining was performed with unconjugated anti-RANKL mAb (clone 70513, R&D Systems) followed by secondary APC-conjugated goat anti-mouse antibody (Becton&Dickinson). This was followed by staining with anti-TCRVα24-FITC (clone C15, Beckman-Coulter), anti-TCRVβ11-PE (Clone C21, Beckman-Coulter) and anti-CD3-PercP (Becton&Dickinson). As isotypic control a second sample was stained in a similar way except that a mock antibody was used instead of anti-RANKL. Data were acquired on a 4-color FACS Calibur cytometer (Becton&Dickinson) and analysis was carried with the CellQuest and FlowJo software. Mean Fluorescence Intensity (MFI) of RANKL and mock antibodies were then calculated in CD3+TCRVα24+Vβ11+iNKT cells and in CD3+TCRVα24-Vβ11- T cells. Results are expressed as ratio of MFI RANKL/MFI mock in CD3+TCRVα24+Vβ11+iNKT cells and in CD3+TCRVα24-Vβ11-T cells.
iNKT cell isolation. PB samples were subjected to Ficoll-Paque™ gradient centrifugation. iNKT cells were purified from PB mononuclear cells after positive selection using the positive selection anti-iNKT cell microbeads kit (anti-TCRVα24Jα18 6B11 mAb, Miltenyi Biotec). Purity was checked in negative and positive fractions by staining with anti-CD3 and anti-TCRVα24Jα18-FITC. Median iNKT cell purity was 87% (range 65-96%).
T-cell activation with anti-CD3/CD28-coated beads. PB mononuclear cells were subjected to negative selection of T-cells, in order to avoid T-cell activation through CD3 engagement using a pan-T cell negative selection kit (Miltenyi Biotec) according to the manufacturer’s instructions. 4x10⁶ T-cells were plated in 1ml of culture medium (RPMI 1640 + 5% Human Serum + Penicillin/Streptomycin + L-glutamine) and activated with 20µl/ml of anti-CD3/CD28-coated beads (Invitrogen) in flat bottom 24-well plates. As a negative control T-cells without anti-CD3/CD28-coated beads were used. At 12 and/or 36 hours samples from activated and non-activated T-cells were obtained and stained as described above.
Real-time PCR (RQ-PCR). Total RNA was extracted from purified iNKT cells and iNKT cell-depleted T-cells using the mini-RNAeasy extraction kit (Qiagen). 0.5-1 µg of total RNA was reversed transcribed to cDNA using the RETRO-Script Kit (Ambion). Taqman assays for quantitative real-time PCR were used to measure mRNA RANKL (encompassing both surface and secreted form; Assay Hs01092186_m1, Applied-Biosystem); 18s rRNA was used as reference gene. The Light-Cycler amplifier (Biorad) with Opticon-3 analysis software was used for data analysis. Expression of RANKL mRNA in purified iNKT cells after its normalization against 18s expression was calculated relative to iNKT cell-depleted T-cells from the same sample and time point. Bars represents the RANKL mRNA of iNKT cells and of T-cells as calculated by the 2^ ΔΔCt method.

## Slide 2
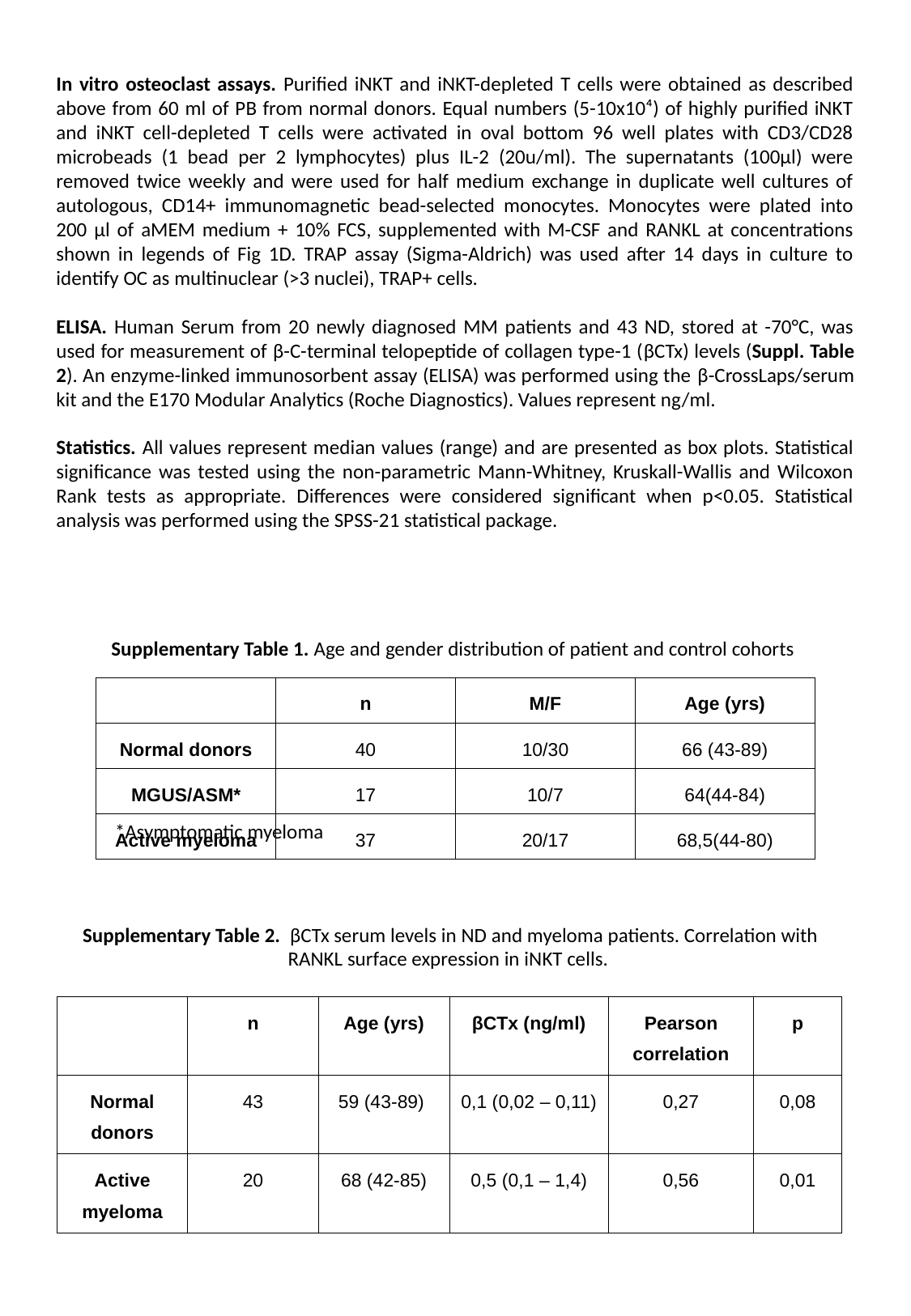

In vitro osteoclast assays. Purified iNKT and iNKT-depleted T cells were obtained as described above from 60 ml of PB from normal donors. Equal numbers (5-10x10⁴) of highly purified iNKT and iNKT cell-depleted T cells were activated in oval bottom 96 well plates with CD3/CD28 microbeads (1 bead per 2 lymphocytes) plus IL-2 (20u/ml). The supernatants (100µl) were removed twice weekly and were used for half medium exchange in duplicate well cultures of autologous, CD14+ immunomagnetic bead-selected monocytes. Monocytes were plated into 200 µl of aMEM medium + 10% FCS, supplemented with M-CSF and RANKL at concentrations shown in legends of Fig 1D. TRAP assay (Sigma-Aldrich) was used after 14 days in culture to identify OC as multinuclear (>3 nuclei), TRAP+ cells.
ELISA. Human Serum from 20 newly diagnosed MM patients and 43 ND, stored at -70°C, was used for measurement of β-C-terminal telopeptide of collagen type-1 (βCTx) levels (Suppl. Table 2). An enzyme-linked immunosorbent assay (ELISA) was performed using the β-CrossLaps/serum kit and the E170 Modular Analytics (Roche Diagnostics). Values represent ng/ml.
Statistics. All values represent median values (range) and are presented as box plots. Statistical significance was tested using the non-parametric Mann-Whitney, Kruskall-Wallis and Wilcoxon Rank tests as appropriate. Differences were considered significant when p<0.05. Statistical analysis was performed using the SPSS-21 statistical package.
Supplementary Table 1. Age and gender distribution of patient and control cohorts
| | n | M/F | Age (yrs) |
| --- | --- | --- | --- |
| Normal donors | 40 | 10/30 | 66 (43-89) |
| MGUS/ASM\* | 17 | 10/7 | 64(44-84) |
| Active myeloma | 37 | 20/17 | 68,5(44-80) |
*Asymptomatic myeloma
Supplementary Table 2. βCTx serum levels in ND and myeloma patients. Correlation with RANKL surface expression in iNKT cells.
| | n | Age (yrs) | βCTx (ng/ml) | Pearson correlation | p |
| --- | --- | --- | --- | --- | --- |
| Normal donors | 43 | 59 (43-89) | 0,1 (0,02 – 0,11) | 0,27 | 0,08 |
| Active myeloma | 20 | 68 (42-85) | 0,5 (0,1 – 1,4) | 0,56 | 0,01 |

## Slide 3
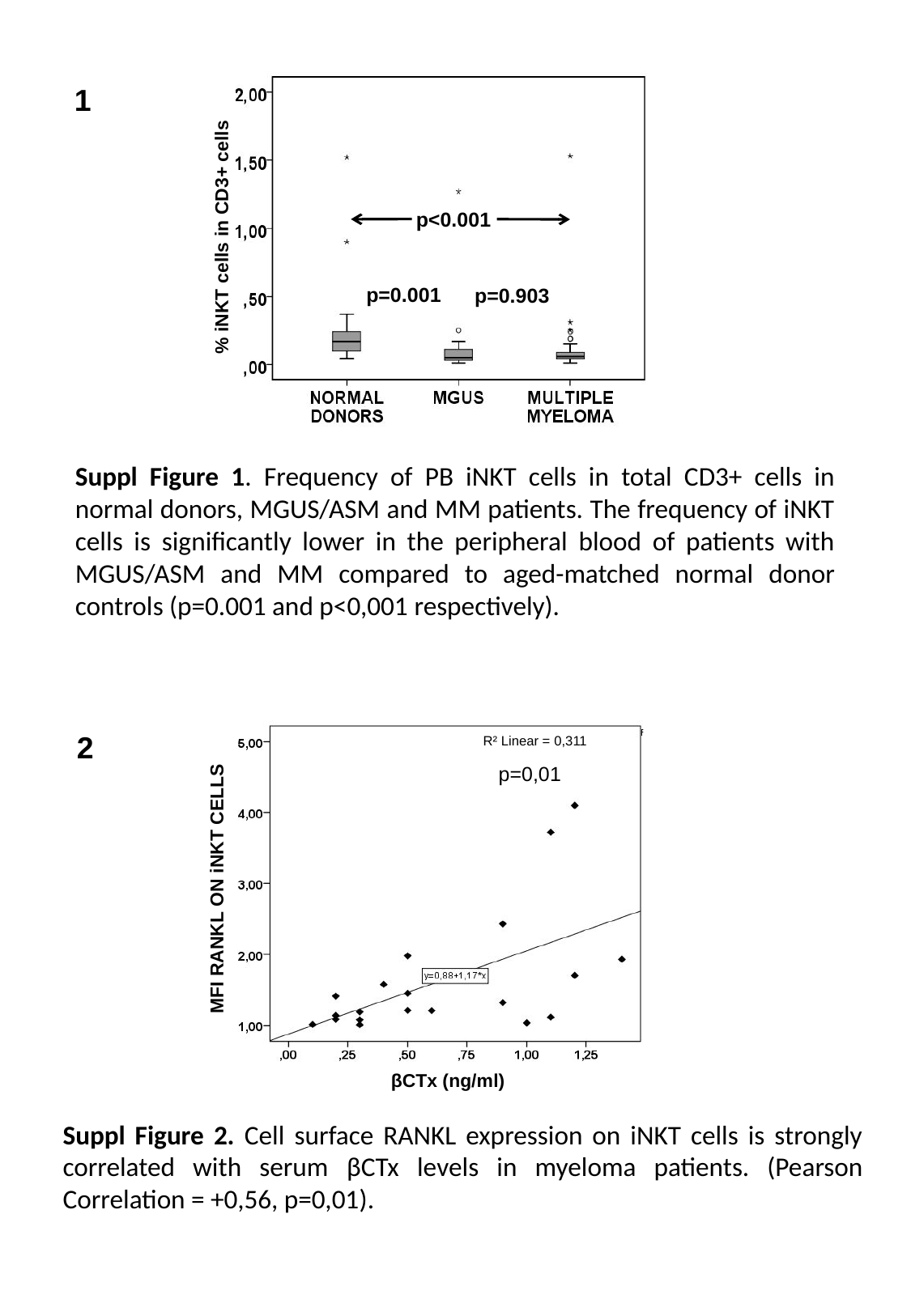

p<0.001
% iNKT cells in CD3+ cells
p=0.001
p=0.903
1
Suppl Figure 1. Frequency of PB iNKT cells in total CD3+ cells in normal donors, MGUS/ASM and MM patients. The frequency of iNKT cells is significantly lower in the peripheral blood of patients with MGUS/ASM and MM compared to aged-matched normal donor controls (p=0.001 and p<0,001 respectively).
R² Linear = 0,311
p=0,01
MFI RANKL ON iNKT CELLS
βCTx (ng/ml)
2
Suppl Figure 2. Cell surface RANKL expression on iNKT cells is strongly correlated with serum βCTx levels in myeloma patients. (Pearson Correlation = +0,56, p=0,01).

## Slide 4
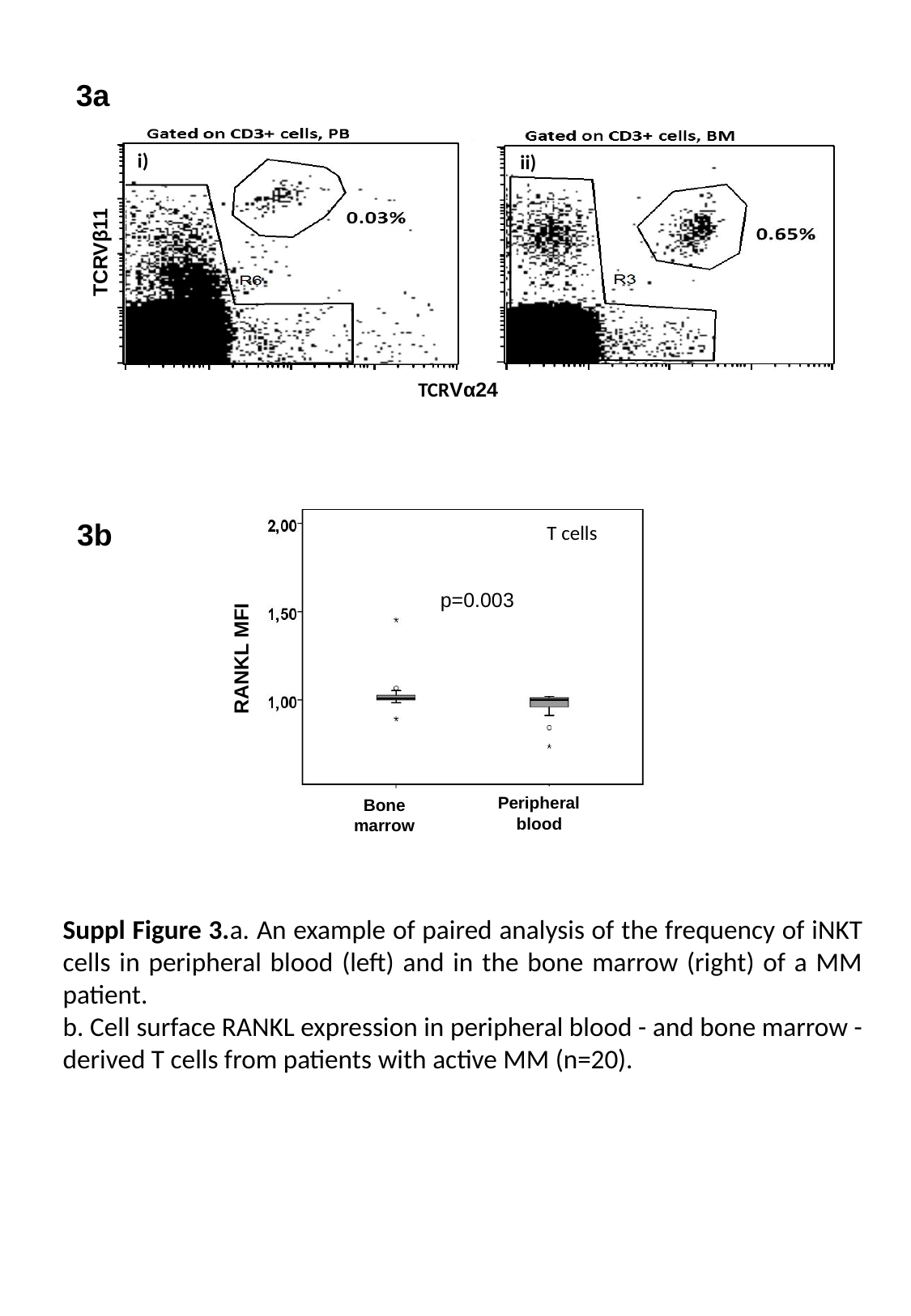

3a
i)
ii)
 TCRVβ11
% iNKT cells
 TCRVα24
T cells
p=0.003
 RANKL MFI
Peripheral
 blood
 Bone
 marrow
3b
Suppl Figure 3.a. An example of paired analysis of the frequency of iNKT cells in peripheral blood (left) and in the bone marrow (right) of a MM patient.
b. Cell surface RANKL expression in peripheral blood - and bone marrow -derived T cells from patients with active MM (n=20).
